# Development and Feasibility Testing of Video Home Based Telerehabilitation for Stroke Survivors in Resource Limited Settings

**DOI:** 10.5195/ijt.2020.6321

**Published:** 2020-12-08

**Authors:** Marufat O. Odetunde, Overcomer T. Binuyo, Fatai A. Maruf, Sunday O. Ayenowowon, Adaobi M. Okonji, Nurain A. Odetunde, Chidozie E. Mbada

**Affiliations:** 1 Department of Medical Rehabilitation, Faculty of Basic Medical Sciences, College of Health Sciences, Obafemi Awolowo University, Ile-Ife, Osun State, Nigeria; 2 Department of Medical Rehabilitation, Faculty of Health Sciences and Technology, Nnamdi Azikiwe University, Nnewi Campus, Anambra State, Nigeria.; 3 Department of Physiotherapy, Obafemi Awolowo University Teaching Hospital, Ile-Ife, Osun State, Nigeria; 4 General Out-Patient Department, General Hospital, Gusau, Zamfara State, Nigeria

**Keywords:** Stroke, Telerehabilitation, Video-based exercises, Yoruba language

## Abstract

Tele-physiotherapy has been shown to be valuable to improve clinical outcomes after stroke. Yet, home-based interventions for stroke survivors (SSVs) who speak indigenous African languages are sparse. This study developed a video-based home exercise programme (VHEP) for SSV speakers of Yoruba. A qualitative descriptive pilot study was conducted in two phases: development and feasibility testing. VHEP development followed the American Stroke Association's recommendations to include demonstrations of task-specific mobility-task and postural training; trunk exercises, and overground walking. The exercise instructions were presented in the Yoruba language. Each exercise was demonstrated for five minutes on video for a total of 30 minutes. The feasibility testing involved ten consenting chronic SSVs. Each imitated the VHEP twice per week for two weeks and thereafter completed a feasibility questionnaire. Criteria for feasibility were: cost of using VHEP, recruitment rate, retention of participants, adherence to the exercises, and intervention delivery. The ten SSVs were recruited within one week, had prior home access to a video player at no-cost, adhered to the exercises as recorded, completed the 30 minute-duration for two weeks, and confirmed intervention delivery of VHEP. Most participants liked the novel use of Yoruba as the language of instruction on VHEP. The VHEP was feasible and acceptable among the studied sample of SSVs. Video based home telerehabilitation for SSVs therefore has the potential to meet the growing need for tele-physiotherapy in resource limited settings.

Stroke is one of the most disabling adult chronic non-communicable conditions and the second leading cause of death worldwide (Donkor, 2018). With an annual mortality rate of about 5.5 million low- and middle-income countries (LMIC) account for nearly 85% of global deaths from stroke (Billinger et al., 2014; Feigin et al., 2013). Stroke constitutes a huge public health burden, estimated to rise over future decades because of the demographic transitions of populations (e.g., increase in population despite endemic poverty and unemployment), especially in developing countries (Donkor, 2018). Stroke incidence is rising in black Africans and other LMIC where community-based studies revealed an age-standardized annual stroke incidence and prevalence rates of up to 316 and 918 per 100,000 population respectively (Owolabi et al., 2015).

Stroke presents with debilitating initial symptoms leading to motor, sensory, perceptual, or cognitive deficits, in addition to environmental and personal factors, all of which can culminate in long-term disability (Chen & Rimmer, 2011). Consequently, stroke rehabilitation requires a long-term multidisciplinary approach. The substantial gains made during in-patient stroke rehabilitation may be followed by an equally substantial decline within six months after discharge (Franck et al., 2017). This necessitates out-patient therapy sessions for stroke survivors (SSVs). Clinicians are faced with the challenge of selecting the most effective ways to sustain the immediate post-intervention outcomes (Altman, 2009; Chen et al., 2011). One of the emerging ways in which physiotherapists are striving to preserve outcomes is to prescribe home exercise programs to SSVs.

The high burden and inadequate rehabilitation services of stroke has brought about the need to develop and evaluate new strategies such as the use of telerehabilitation. Telerehabilitation is a term used to describe the provision of rehabilitation and habilitation services to adults and children at a distance via information and communications technologies (ICTs) (Russell, 2007; Richmond et al., 2017). Clinically, telerehabilitation can include assessment, monitoring, prevention, intervention, supervision, education, consultation, and counseling (Richmond et al., 2017). This method of rehabilitation service delivery is suitable for patients who live at a distance and therefore find it difficult to attend clinic regularly due to the time and cost of travel Adhikari et al., 2020; Odole et al., 2015).

Though tele-physiotherapy has been proven effective for several medical conditions in different parts of the world, its usage by physiotherapists in Nigeria is low. This is due to cultural barriers and financial factors (Odole et al., 2015). Notwithstanding, some studies in Nigeria have shown the effects of tele-physiotherapy on orthopedic conditions such as knee osteoarthritis (Adhikari et al., 2020; Odole et al., 2014) and low back pain (Adhikari et al., 2020; Mbada et al., 2019). Home programming administered through telerehabilitation is promising in sub-Saharan Africa (SSA) and may be valuable to improve quality of life and clinical outcomes after stroke (Linder et al., 2015; Sarfo et al., 2018). In resource limited settings like Nigeria, SSVs are usually home bound and restricted in physical activities due to inadequate and unaffordable rehabilitation services. To our knowledge, there are no video-based rehabilitation interventions that use improvised indigenous exercise apparatus and indigenous language for SSVs in African countries.

The objective of this study was, therefore, to develop a video-based home exercise programme (VHEP) for SSV in the English and Yoruba languages and conduct feasibility testing of the programme. Yoruba is the indigenous language of the people of southwestern Nigeria spoken by about 17 million people in Nigeria (National Population Commission [NPC], 2006) and up to 13 million people in the neighboring countries of the Republic of Benin and Togo.

Criteria for feasibility of this study were: cost of using the VHEP by the SSVs, recruitment rate, retention of participants, adherence to the exercise, and intervention delivery.

## MATERIALS AND METHODS

The study is a qualitative descriptive pilot study divided into two phases: development and feasibility testing.

### DEVELOPMENT PHASE

#### PARTICIPANTS

A 55-year old consenting male chronic SSV served as the videotaped exemplar for the development of the VHEP. His stroke severity was mild (Modified Rankin Scale [mRS] score of 2) and independent in ADLs (Modified Barthel Index [mBI] score of 17). Modified Ashworth scale (MAS) score was 1+ and Brunnstrom Stage of Motor Recovery was 4. Vital signs were within normal limits. Exercise intensity of 70% of maximal heart rate was determined for the participant (Pang et al., 2006) while his level of exertion was monitored via a modified 11-point Borg Rating of Perceived Exertion (RPE) Scale during the intervention.

#### METHODS

The video-based home exercises in this study followed the evidence-based exercise recommendations for SSVs by the American Heart Association/American Stroke Association (AHA/ASA) in 2016 (Winstein et al., 2016). These recommendations include: repetitive, task-specific training for upper extremity function; intensive, repetitive, mobility-task training; and overground walking exercise training combined with conventional rehabilitation, postural training, and trunk exercises (Winstein et al., 2016). The instructions and descriptions of each of the exercises were written in a script in English language. A translated Yoruba version of the script was produced by a secondary school teacher of Yoruba language and reviewed by a Yoruba physiotherapist for equivalence. The videotaped SSV demonstrated the exercises under the direction of an instructor while the digital technician recorded the exercise programme into a video. Each exercise was demonstrated for five minutes with rest period of one minute between exercises for a total duration of thirty minutes.

### FEASIBILITY TESTING PHASE

#### PARTICIPANTS

Stroke survivors attending the out-patient physiotherapy clinic of the Obafemi Awolowo University Teaching Hospital Complex (OAUTHC), Ile-Ife, Nigeria were selected for feasibility testing of the VHEP. Participants were included if they had stroke three or more months ago, presented with hemiplegia or hemiparesis, and had Modified Ashworth scale (MAS) scores of 1 or more and Brunnstrom stage 3 and above. SSVs with psychiatric illness, epilepsy, metastatic cancer, uncontrolled blood pressure, diabetes mellitus, an underlying cardiac condition that could be aggravated by exercise, other associated co-morbidities, and dependence on a caregiver or on hospital admission were excluded from this study. Sample size calculation for feasibility testing was as per Sauro (2011): Log (1-X)/Log (1-Y) where X =percentage chance of detecting a problem with the video and Y is probability of occurrence of a problem. The authors suggested 80%, 85%, 90% or 99% chance of detecting a problem. More obvious problems (10-30%) require less sample size while less obvious problems (1%) require more sample size, and a lot of time and budget. This study sought to see the more obvious problems on the video that affect 1/3rd or more users with an 85% chance of seeing them. Therefore, X was put at 85% and Y at 33%; Log (1-0.85)/log (1-0.33) = 4.73. A minimum of five users were to be tested. To allow for possible drop out from the study, and considering that the calculated sample was small, a sample size of 10 was proposed. This is consistent with a stroke-specific study by Sheehan et al. (2006) that had recommended a sample size of up to 12 for pilot and feasibility studies among SSVs.

#### INSTRUMENTS

A Feasibility Questionnaire was adapted from the Satisfaction Survey for the Individual with Stroke in a study on a smartphone-enabled, educational intervention conducted by Sureshkumar et al. (2015). Feasibility and acceptability were assessed through a semi-structured questionnaire. Eighteen relevant items were extracted from the 41-items on the Satisfaction Survey Assessment form for the Individual with Stroke section of the Sureshkumar et al. (2015) questionnaire. The Satisfaction Survey Assessment form for Caregivers of Individual with Stroke was excluded. The items were adapted by replacing ‘smartphone' with ‘video' for use in the current study. The 18 items contained both open and closed-ended questions taken from the three sections. These assessed orienting and training of participants to the intervention, content of the intervention, and utilization of the intervention administered to stroke survivors. The closed-ended questions included ordered (Likert scale) responses. Participants were also asked specific open-ended questions related to the objectives of the feasibility testing. Demographic and clinical details of the stroke survivors were also obtained.

The Modified Borg Rating Scale of Perceived Exertion (RPE) is a patient reported outcome tool used to measure a person's perception of their effort and exertion, breathlessness, and fatigue during exercise. The modified RPE is 11-point scale with verbal description that ranges from 0=no exertion at all to 10= very, very hard. The tool is recommended for use in sub-acute (2-6 months) and chronic (> 6 months) SSVs (Shirley, 2018).

Modified Rankin Scale: This is a 6-point clinician-reported measure of global disability after a stroke or other causes of neurological disability. It ranges from 0= No disability to 5= Severe disability. A separate category of 6 is added for patients who are deceased.

### PROCEDURE

Ethical approval was obtained before the commencement of this study. The purpose of the research was explained to the participants and their written informed consent was obtained prior to their taking part in the study. Each of the ten SSVs underwent a pre-treatment assessment of level of disability using the Modified Rankin Scale and measurement of blood pressure. Heart rate and respiratory rate with exercise intensity of 70% of maximal heart rate was determined for each participant (Pang et al., 2006).

The video incorporated personalized and guided self-therapy using the recommendations of AHA/ASA (2016). However, it should be noted that the video is exclusively a product of the authors and was not developed in concert with the AHA/ASA. The videos began with an introduction, followed by five short exercises in different positions (i.e., reclining, kneeling, sitting, standing, and walking). The exercises emphasized repetition, gradually progressive task difficulty, and functional practice at a self-selected speed. Each exercise intervention was demonstrated for five minutes with a rest period of one minute between exercises, and all within a total duration of 30 minutes (Dettmers et al., 2014). Participants' exertion rates were monitored throughout the intervention using their feedback from the modified RPE.

The video was played for each of the 10 stroke survivors individually. The participants imitated the exercises twice in a week for two weeks after which they completed the feasibility questionnaire. No undue level of exertion was reported by any of the participants throughout the duration of the intervention.

**Figure 1:**
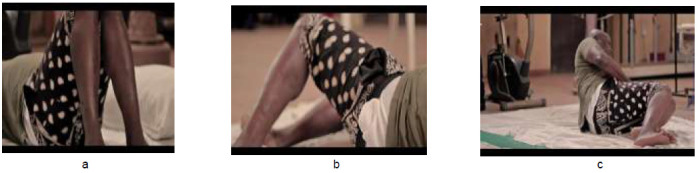
Graded Exercises from (a) Supine (b) to Lateral Trunk Rotation (c) to Side Lying

**Figure 2:**
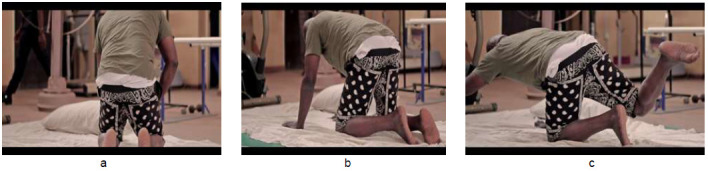
Graded Exercises from (a) High Kneeling (b) to Four-Point Kneeling (c) to Alternate Limb Raising/Lowering in Four-Point Kneeling Position

**Figure 3:**
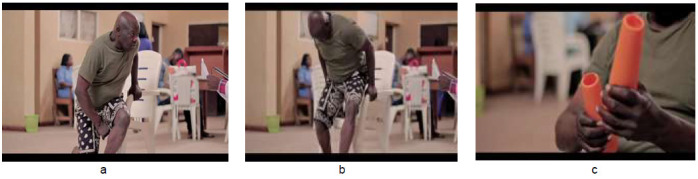
Transfer from (a) Four-point Kneeling to High Kneeling with Chair Support (b) Transfer into Sitting on a Chair (c) and Repetitive Picking and Placing a Cup from a Pack from the Top to Bottom

**Figure 4:**
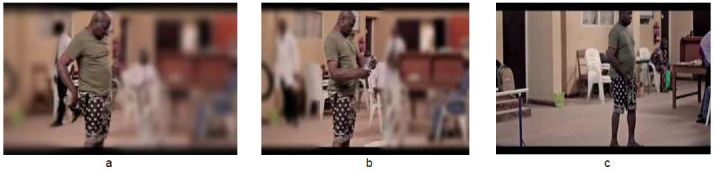
Standing Position, Repetitive Task Training (a) Posteriorly (b) Anteriorly and (c) Four Sided Walking: Four Steps Each in the Forward-Backward-Right-Left Sides

The exercises were demonstrated as shown in figures 1 to 4. Figure 1 depicts graded exercises from (a) supine (b) to lateral trunk rotation (c) to side lying. Figure 2 shows graded exercises from (a) high kneeling (b) to four-point kneeling (c) to alternate limb raising/lowering in four-point kneeling position. Figure 3 involves transfer from (a) four-point kneeling to high kneeling with chair support (b) transfer into sitting on a chair (c) and repetitive picking and placing a cup from a pack from the top to bottom. Figure 4 depicts exercises in standing position, alternate picking-up and passing a bottle of water from (a) one hand to the other (b) through the back; and (c) four sided walking: four steps each in the forward-backwards-right-left sides. Each exercise was demonstrated for five minutes once the patient assumed the desired position, followed by a rest period of one minute with deep breathing exercises between one exercise and the next. The total duration of the exercises was approximately thirty minutes.

## DATA ANALYSIS

Descriptive statistics of mean, standard deviation, frequency and percentages was used to summarize demographic and clinical characteristics as well as present participants' responses using SPSS version 22.0 (Chicago IL SPSS Inc.).

### RESULTS

Participants in this feasibility study consisted of 10 SSVs (5 males and 5 females). The mean age of participants in this study was 59.20±7.40 with age ranging from 47 to 70 years. Mean time since a stroke was 23±5.6 months. All participants had an ischemic stroke of varying severity (MRS score 2 to 4). Other demographic details of participants are presented in Table 1.

**Table 1:** Socio-demographic and Clinical Characteristics of Participants

Variables	Frequency	Percentages (%)
**Socio-Demographic**		
**Gender**		
Male	5	50
Female	5	50
**Age (years)**		
50 and below	2	20
51-60	2	20
61-70	5	50
**Education**		
No formal education	1	10
Primary school	3	30
Secondary school	2	20
Tertiary education	4	40
**Current occupation**		
Daily wage labour	1	10
Small scale business	4	40
Not working	5	50
**Clinical**		
**Stroke type**		
Ischemic	10	100
Hemorrhagic	0	0
**Stroke severity**		
Mild	4	40
Moderate	4	40
Severe	2	20
**Affected side**		
Left	6	60
Right	4	40

All criteria for feasibility were met: the cost of using VHEP, a robust recruitment rate, participant retention, adherence to the exercises, and intervention delivery. The ten eligible SSVs were recruited within one week, completed the two week-duration of the VHEP, and adhered to the exercises. All participants reported that the VHEP was well delivered and carried out.

Table 2 shows the experience of participants on the usage of the video player. All participants owned a video player which they had been using for the mean duration of 11.6±9.4 years prior to having a stroke. Therefore, they incurred no equipment costs. Half required the assistance of a caregiver to operate the equipment.

**Table 2:** Experience of Participants and Caregivers on the Video Player Usage

Variables	Responses	Percentages (%)
Experience of SSV on use of video player	Yes	80
	No	20
Experience of CG on the use of video player	Yes	90
	No	10
Does the SSV own a video player?	Yes	100
	No	0
Does the CG own a video player?	Yes	90
	No	10

*Note.* Key: SSV - stroke survivors that are participants; CG - caregivers

Questions on orientation of participants to the VHEP showed that their initial impression of the intervention was good for most (90%) (Table 3).

**Table 3:** Initial Impression and Feeling When Informed About the Video Intervention

Variables		Frequency (n)	Percentage (%)
Initial impression about video intervention	Good	9	90
	Bad	1	10
Feelings when told about the intervention	Hopeful	2	20
	Happy	5	50
	Indifferent	3	30

All participants said they received adequate information and had confidence in performing the contents of the video before beginning the VHEP (Table 4).

**Table 4:** Instructions and Demonstrations Received Before Beginning the Program

Variables	Responses	Frequency (n)	Percentages (%)
Need help	Caregivers	5	50
	Both caregiver and training	3	30
	Manage oneself	2	20
Sufficient information	Yes definitely	6	60
		4	40
	No		
Clear instructions	Yes definitely	5	50
	Yes, to some extent	5	50
Clear demonstrations	Yes definitely	5	50
	Yes, to some extent	5	50
Enough confidence	Yes definitely	4	40
	Yes, to some extent	6	60

The participants all affirmed that the video information was presented in an understandable manner and that the intervention was relevant to their current needs. Most participants found exercises performed on the mat (40%) more interesting, and exercises when standing (50%) less interesting (Table 5).

**Table 5:** Content of the Intervention

Questions	Responses	Frequency (n)	Percentage (%)
Do you think that the video information was presented in a way you could watch and understand?	Yes, definitely	4	40
	Yes to some extent	6	60
	No	0	0
Were the intervention videos relevant to your current needs?	Yes completely	5	50
	Yes to some extent	5	50
	No	0	0
Which section was more interesting?	Mat exercises	4	40
	Exercises on chair	3	30
	Exercises in standing	2	20
	Exercises in walking	1	10
Which section was less interesting?	Mat exercises	2	20
	Exercises on chair	1	10
	Exercises in standing	5	50
	Exercises in walking	2	20

Table 6 shows the utilization of the intervention by the participants. Most of the participants (60%) opined that the video was useful for general body impairment and 40% liked the goal orientation of VHEP. Sixty percent reported that the use of Yoruba as the language of instruction of the VHEP was novel.

**Table 6:** Utilization of the Intervention by Participants

Variables	Reponses	Frequency (n)	Percentage (%)
Those who watched the video with you	Fellow patients	5	50
	Caregiver	3	30
	Nobody	2	20
Ways the video was useful	General body improvement	6	60
	Psychological euphoria	1	10
	More mobility	3	30
Things liked about the intervention	Goal oriented	4	40
	Different segment covered	2	20
	Simple to perform	4	40
Anything new in this video	the Yoruba language	6	60
	the good introduction	1	10
	Nothing new	3	30
How they will demonstrate the activities	Simply follow instructions	7	70
	Contacting the instructor	1	10
	No demonstration of home program	2	20
Will the video be useful to other stroke survivors	Yes definitely	6	60
	Yes probably	4	40

## DISCUSSION

Early studies on the feasibility and effectiveness of video-based treatment showed that patients hold positive attitudes toward video-based rehabilitation (Fernández-González et al., 2019; Kubota et al., 2013). In the current study, participants displayed good orientation and expressed happiness when informed about the VHEP. This led to rapid and high recruitment rate of SSVs, even above the minimum number of required sample within a short period.

Feasibility testing of the VHEP provided an opportunity to review and revise the intervention before taking it through validation on a larger sample of stroke survivors. The feasibility testing revealed that every participant's family had a video player in their homes, at no additional cost. This suggests that VHEP is potentially accessible in resource limited settings in which expensive telecommunication technology may not be available for tele-physiotherapy. Although all participants owned video players they had been operating before having a stroke, half required help from caregivers to operate the video.

The investigators relied heavily on feedback from the SSVs. It is encouraging that participants found the intervention to be relevant to their needs. The SSVs positively viewed the VHEP protocol. They opined that it was goal oriented and simple to perform. Validating their views, is that none of the 10 SSV defaulted throughout the two week-duration of the VHEP. All adhered to the exercises which they found easy to understand and complete by simply following the instructions on the video. Most SSVs found the VHEP especially useful to address general body impairment and believed that the intervention will be useful for other SSVs. This implies a high level of acceptability of the intervention, similar to the report of Sureshkumar et al. (2015).

The new and exciting attribute of the video for most respondents was the use of Yoruba language as the medium of instruction. Considering the non-availability and inaccessibility of this type of home-based rehabilitation exercises in indigenous language communities, VHEP has the potential to fulfill an important need for SSVs.

Some cautions are in order. More than half of the stroke survivors were confident only to some extent in using the intervention. This may be why most of the stroke survivors found exercises on the mat more interesting and exercises that required standing least interesting. It appears that the more interesting exercises were the ones participants found safer and easier to do. This may call for need to emphasize caution in the form of instructions in these exercises. Although VHEP may be a practical link that may help mediate the challenges of barriers of distance, time, and travel to receive care, further emphasis should also be placed on the assertion that it will not completely replace the traditional in-person interaction with a health-care professional (Mbada et. 2019; Vardeh et al, 2013).

This intervention accommodated chronic SSVs with mild to moderate physical disability. Future studies should validate the VHEP in a larger sample of SSVs and assess efficacy of the intervention on the relevant clinical outcomes. Similar interventions should be developed for acute and sub-acute SSVs. The VHEP might also be adapted for other indigenous languages.

## CONCLUSION

Development and feasibility testing of VHEP revealed that it is feasible, acceptable, and useful among a sample of SSVs with mild to moderate stroke in a Nigerian context. This suggests that provision of a video-based home exercise intervention with instructions in the Yoruba language for management of post-stroke disabilities could be a potential strategy to meet the growing need for stroke rehabilitation services in resource limited settings.
